# Supera cognitive stimulation study with cognitively-unimpaired older adults: methodology and initial results of a randomized controlled clinical trial

**DOI:** 10.1055/s-0045-1809882

**Published:** 2025-07-02

**Authors:** Thais Bento Lima da Silva, Tiago Nascimento Ordonez, Gabriela dos Santos, Laydiane Alves Costa, Ana Paula Bagli Moreira, Diana dos Santos Bacelar, Maria Antonia Antunes de Souza, Sabrina Aparecida da Silva, Sonia Maria Dozzi Brucki, Monica Sanches Yassuda

**Affiliations:** 1 Universidade de São Paulo, Escola de Artes, Ciências e Humanidades, Programa de Pós-Graduação em Gerontologia, São Paulo SP, Brazil.; 2 Universidade de São Paulo, Faculdade de Medicina, Departamento de Neurologia, Divisão de Clínica Neurológica, Grupo de Neurologia Cognitiva e do Comportamento, São Paulo SP, Brazil.

**Keywords:** Cognition, Surveys and Questionnaires, Aged, Aging

## Abstract

Scientific investigations have highlighted the benefits of cognitive stimulation for cognitive, psychological, and social aspects in older individuals. However, there is a dearth of long-term, methodologically-rigorous studies. The aim of the present study was to describe the methods and the initial characteristics of the participants in a randomized controlled trial on cognitive stimulation. A total of 578 older individuals accepted invitations to participate in the study. Of these respondents, 362 met the eligibility criteria, and 255 were selected and randomized into the training, active control, and passive control groups. During the baseline stage (T0), 48 participants withdrew, resulting in a final T0 sample of 207 participants. The three groups were similar in terms of cognitive performance and sociodemographic and psychosocial variables, but they differed significantly regarding depressive symptoms, with the training group scoring higher. The methods herein described can help guide future research on cognitive stimulation in older adults.

## INTRODUCTION

Brain plasticity refers to the brain's capacity to form new neuronal connections in response to individual needs and the environment. 1 Cognitive stimulation seeks to activate higher cognitive functions, such as memory, reasoning, language, and executive functions, employing different resources to slow cognitive decline. Encouraging older individuals to engage in cognitively-stimulating activities has become increasingly important, with the goal of enhancing their quality of life and, as far as possible, preserve and improve brain function. 2

Many cognitive interventions have been reported in the literature. 3 Gavelin et al. 2 identified three main types: cognitive training, cognitive rehabilitation, and cognitive stimulation. Cognitive stimulation encompasses a variety of activities aimed at preserving overall cognitive function while encouraging mental and social engagement. This type of intervention is recommended for older adults with mild-to-moderate dementia, as well as for cognitively-unimpaired individuals. These activities can be performed individually, but also in social and community settings. The present article focuses on cognitive stimulation.

Reviews and meta-analyses have demonstrated the benefits of cognitive stimulation for cognitive domains 4 as well as psychosocial aspects, through improvements in quality of life and reductions in depression and anxiety. 5 These psychosocial variables are strongly linked to cognitive performance, making it essential to maintain or improve these aspects in older adults with or without cognitive impairment. 4 Calatayud et al. 6 conducted a clinical trial involving 201 volunteers, and the results showed that the intervention group presented better cognitive performance than the control group after 10 sessions and at subsequent evaluations.

Previous studies had certain limitations, such as a lack of randomization and blinding of evaluators, 3 reinforcing the need for further research with postintervention follow-up, particularly in Brazil. Such studies can demonstrate the impact of strategies that promote cognitive health, preserve the autonomy of older adults, and reduce the burden on the social and healthcare systems. 7

Despite the growing importance of cognitive stimulation, there is a dearth of robust studies in the literature on the efficacy of this strategy. 8 There are some challenges in identifying the reliability of results for cognitive intervention studies, such as a lack of methodological vigor, small samples, and the heterogeneity of the interventions, with most studies involving multiple intervention components besides cognitive stimulation. 9

Other researchers have described the methodological procedures of studies involving cognitive interventions. 10 11 12 13 In this respect, Zülke et al. 14 described the protocol of a study whose objective was to develop a prevention program against cognitive decline in older community-dwelling adults. Yoon et al. 15 reported the methodological characteristics of a cognitive training study in adults, together with results at baseline, including sociodemographic information on the sample. More recently, Crivelli et al. 10 reported the study design and harmonization strategies of the Latin American Initiative for Lifestyle Intervention to Prevent Cognitive Decline, providing the sociodemographic data and health status for the sample. These studies highlight the importance of describing the design of randomized controlled trials, most notably to enable reproducibility.

The objective of the Supera Cognitive Stimulation Study clinical trial was to investigate the impact of the Supera cognitive stimulation program on cognitive performance and psychosocial variables in healthy older adults. In the present article, a description of the clinical trial design is provided, along with the initial results collected at baseline between February and March 2022.

## METHODS

### Study overview

**Figure 1:**
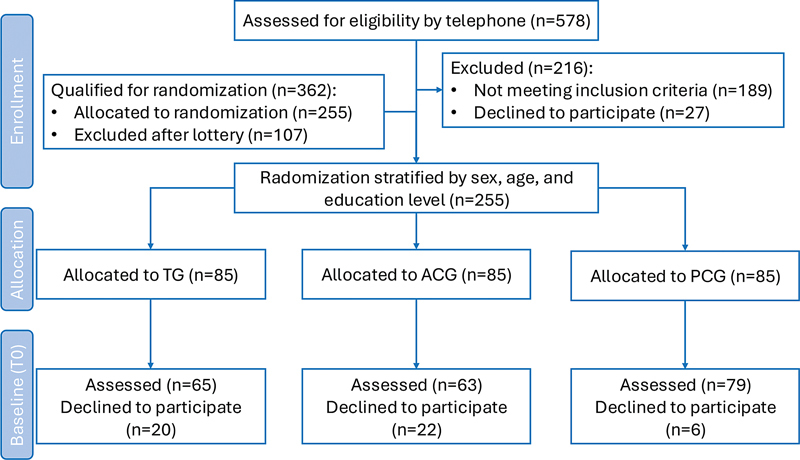
Diagram of the Consolidated Standards of Reporting Trials (CONSORT) statement.

The baseline assessments before randomization (T0) have been completed ( Figure 1 ), while the subsequent assessments will be conducted at 6 (T1), 12 (T2), and 18 months (T3) throughout the intervention. Finally, a follow-up assessment will be conducted 24 months postintervention (T4).

### Sample selection

After publicizing the study at community centers and retiree associations in the city of São Paulo, Brazil, the study was advertised on social media platforms. The recruitment strategies included information posters, registration forms, telephone and in-person contact with retirees' associations, clubs, and cultural centers for older adults. The persons interested in participating contacted the research assistants by telephone or e-mail. The research assistants enrolled all voluntary participants for the cognitive screening and concealed their identification data using sequential coding.

However, of this initial group, 28 participants withdrew and failed to complete the process, while a further 188 were excluded due to the presence of symptoms of depression (n = 56), anxiety (n = 56), suspicion of or possible dementia (n = 2), functional impairment (n = 22), and previous participation in cognitive stimulation programs (n = 52).

The sample size was calculated, and the funding constraints, considered. A total of 362 subjects met the eligibility criteria, 255 of whom were randomly selected. Of this group, 207 agreed to participate in the study at baseline. All participants took part in the study on a voluntary basis, and the program, materials and assessments were all provided free of charge. This information was stated in the Free and Informed Consent Form (FICF) signed by all participants and researchers involved.

### Randomization

In an effort to reduce the risk of selection bias and ensure comparability across groups, the Excel software (Microsoft Corp.) was used to perform the stratified randomization; 16 initially, the participants were grouped into 8 strata according to sex (male and female), age (older or the same as the median age of the sample and younger than the median) and level of schooling (the same or higher than the median and lower than the median).

For each stratum, a list of random numbers was generated using the “rand.” function, which enabled the participants to be allocated in random order. Based on this order, individuals were randomly assigned into the training group (TG; n = 65), the active control group (ACG; n = 63), and the passive control group (PCG; n = 79).

### Eligibility criteria

The study included participants who were aged ≥ 60 years, had at least a primary level of schooling (4 years of formal education) and scored > 15 on the Brazilian Telephone-Based Mini-Mental State Exam (Braztel-MMSE), which indicates healthy cognitive functioning, as per the specificity and sensitivity criteria established by Camozzato et al. 17 In addition, participants had to score < 6 on the Geriatric Depression Scale (GDS), 18 which indicates absence of depression. Individuals with controlled clinical conditions or in use of medications to manage depressive or anxious symptoms were also deemed eligible, provided that their scores did not exceed the cut-off points for depression (GDS > 5) or anxiety (score > 9 on the Geriatric Anxiety Inventory, GAI). 19 To complement the functional assessment, the participants had to nominate a family member or friend to fill out the Functional Activities Questionnaire (FAQ), 20 with subsequent exclusion of individuals scoring > 2 points on it.

Moreover, individuals who were aged < 60 years, exhibited visual, hearing or motor deficits that affected their ability to perform the cognitive tasks, presented severe psychiatric disorders, clinical or neuroimaging evidence suggestive of vascular problems, or previous diagnosis of dementia, were also excluded. Most of the criteria were analyzed based on information self-reported by the participants.

### Screening protocol and neurological assessment

The screening assessed sociodemographic variables and health conditions using the following instruments: the Sociodemographic Variables Questionnaire, the Lie/Bet Questionnaire (for pathological gambling), 21 the Braztel-MMSE, 17 the FAQ, 20 the GDS, 18 the GAI, 19 the Cut Down, Annoyed, Guilty, and Eye-Opener (CAGE; for alcohol-related issues), 22 and a questionnaire on coronavirus disease 2019 (COVID-19), vaccination, and changes in mood and anxiety.

After applying the exclusion criteria, the selected participants underwent a neuropsychological evaluation using the following instruments: the Addenbrooke's Cognitive Examination-Revised (ACE-R); 23 the Short Cognitive Test Performance (Syndrom Kurztest, SKT); 24 F-A-S Phonemic Verbal Fluency Test (FAS); 25 Digit Span Forward (DSF) and Digit Span Backward (DSB); 26 27 Spatial Span Forward (SSF) and Spatial Span Backward (SSB); 26 28 Letter Number Sequencing; 26 27 the Digit Symbol Substitution Test (DSST); 29 the Trail-Making Test (TMT); 30 and the Abacus Calculation Test. 31

Psychosocial variables were assessed using the following scales: GDS; 18 the Depression, Anxiety, and Stress Scale (DASS); 32 the Control, Autonomy, Self-realization, and Pleasure Scale (CASP-19); 33 the Minimum Map of Relationships of Older Individuals (MMROI); 34 and the Cognitive Function Instrument (CFI). 35 The evaluators were blinded to the participants' experimental condition. The assessment lasted 90 minutes.

### Description of the interventions

#### *Training group*

**Figure 2:**
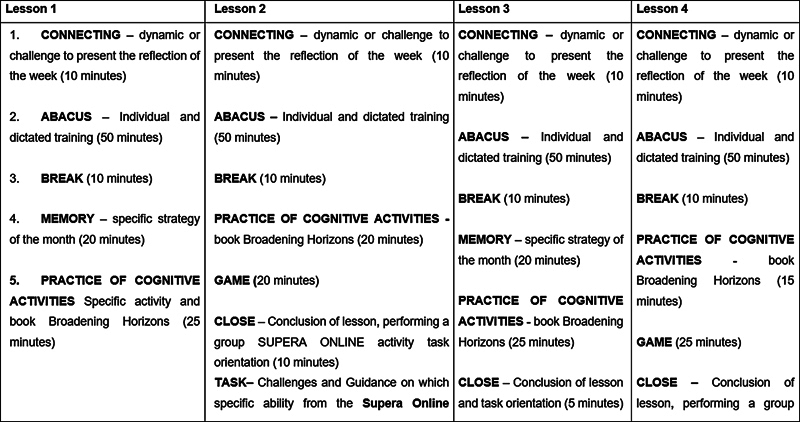
Model of the cognitive sessions.

The TG took part in 72 weekly sessions of 2 hours each ( Figure 2 ). The Supera Stimulation Program has been described in more detail elsewhere. 36 At each session, the attendance of the participants was monitored, with a minimum presence of 80% required for each semester.

A script created by the program instructors outlined the strategies applied to meet the aims of each session, ranging from abacus training for mathematics calculations, folders containing a variety of cognitive exercises (such as “spot the difference”, strategy and memory activities, and visuospatial perception exercises), board games, an online platform with exercises, group dynamics, and “neurobics”.

At the end of each session, the participants were asked to carry out 30 minutes of online exercises using the Supera Online Platform and continue practicing specific skills at home until the next session. Moreover, individual goals were set for abacus-based activities and a folder containing cognitive activities and online activities for completion during the practical sessions and at home was provided.

#### *Active control group (ACG)*

The ACG underwent a gerontological education program with the same duration and intervention time as the TG. The purpose of this control group was to document the benefits of participating in a psychoeducation group and determine whether the gains of the TG exceeded those of the ACG.

Folders were produced and printed containing texts and questions to consolidate each topic studied. In addition, audiovisual materials to illustrate the discussions were also produced. The topics addressed were related to biopsychosocial aspects of aging and late life, promoting health and disease prevention, rights of older people, protagonism of individuals aged ≥ 60, among others.

Similar to the training group (TG), the active control group (ACG) participated in structured sessions guided by pre-defined scripts. These scripts included specific discussion topics, interactive activities to reinforce the session content, estimated time for each component, and reflective exercises assigned as homework. Unlike the TG, however, the ACG sessions focused solely on health and lifestyle education, with no direct emphasis on cognitive stimulation. Thus, the ACG adhered to the same model applied to the TG, and the minimum required attendance rate was of 80% per semester, as in the TG.

#### *Passive control group*

The PCG underwent no intervention of any kind, only the assessment during the same period, as the other groups.

### Ethical aspects

The current study was approved by the Ethics Committee for Research in Humans of Hospital das Clínicas da Faculdade de Medicina da Universidade de São Paulo (HCFMUSP) under no. 4.357.429 and registered at the Brazilian Clinical Trials Registry under identification no. RBR-10wnp828. The present study complied with the guidelines of the Consolidated Standards of Reporting Trials (CONSORT) statement.

In order to avoid any conflict of interest among the parties, and given the study involved a commercially-available cognitive stimulation program, documents declaring no conflict of interest were signed. Moreover, all data were collected and analyzed by independent professionals who were not part of the program.

### Statistical analysis

The sample profile was presented using tables with descriptive statistics, including frequencies, measures of central tendency, and measures of dispersion.

The Kolmogorov-Smirnov's test confirmed that the numeric variables had a non-normal distribution; therefore, non-parametric tests were applied. The Kruskal-Wallis test was employed to compare continuous and ordinal data among the groups. Spearman's correlations were used to analyze the relationships among the variables. The Chi-squared test was employed compare the categorical variables among the groups.

The data were keyed into Google Sheets (Google LLC) and subsequently treated and analyzed using the software applications R (R Foundation for Statistical Computing) and Statistica (TIBCO Software Inc.), version 7.0. The level of significance adopted for the statistical tests was 5% ( *p* < 0.05).

### Procedures

The assessments of the cognitive screening stage were performed by gerontologists, with a discussion about the clinical conditions with the researchers responsible for the current study, a neurologist, a neuropsychologist, and a gerontologist specializing in cognitive and behavioral neurology. The neuropsychological assessment lasted 90 minutes and was performed by neuropsychologists blinded to the experimental conditions of the present study but duly trained for it. The participants signed a FICF and the anonymity and confidentiality of the data at all stages of the study will be guaranteed, as well as the participants' right to withdraw from the study at any time.

## RESULTS

Table 1 shows the sociodemographic variables of the 207 participants, who were randomized as follows: TG – n = 65; ACG – n = 63; and PCG – n = 79. The participants were predominantly of the female sex (73.43%), had a mean age of 67.55 (±5.29) years, and a mean of 17.03 (±4.64) years of schooling. Regarding marital status, 48.79% reported being married. Overall, 85% of the participants stated they were pensioners or retirees. The groups were similar in terms of sociodemographic characteristics ( Table 1 ).

**Table 1:** Sociodemographic characteristics of the study sample

Variable		Total (N = 207)		TG (n = 65)		ACG (n = 63)		PCG (n = 79)		*p-value*
								n		
Sex: n (%)	Female	152 (73.43)		50 (76.92)		47 (74.60)		55 (69.62)		0.595 ^a^
	Male	55	26.57	15	23.08	16	25.40	24	30.38	
Age (years)	Mean(±SD)	67.55(±5.29)		67.48(±5.35)		67.89(±5.89)		67.33(±4.75)		0.954 ^b^
	Median (min.–max.)	67.00 (60.00-89.00)		67.00 (60.00-83.00)		67.00 (60.00-89.00)		67.00 (60.00-84.00)		
Schooling (years)	Mean(±SD)	17.03(±4.64)		16.68(±3.95)		17.60(±5.98)		16.86(±3.91)		0.968 ^b^
	Median (min.–max.)	16.00 (8.00–45.00)		16.00 (9.00–27.00)		16.00 (11.00–45.00)		17.00 (8.00–27.00)		
Marital status: n (%)	Married	101 (48.79)		30 (46.15)		30 (47.62)		41 (51.90)		0.906 ^a^
	Divorced	32 (15.46)		11 (16.92)		8 (12.70)		13(16.46)		
	Single	46 (22.22)		15 (23.08)		17 (26.98)		14 (17.72)		
	Widowed	28 (13.53)		9 (13.85)		8 (12.70)		11 (13.92)		
Retired or pensioner: n (%)	Yes	176 (85.02)		56 (86.15)		54 (85.71)		66 (83.54)		0.894 ^a^
	No	31 (14.98)		9 (13.85)		9 (14.29)		13 (16.46)		

Abbreviations: ACG, active control group; max., maximum; min., minimum; PCG, passive control group; SD, standard deviation; TG, training group.

Notes: ^a^ Chi-squared test; ^b^ Mann-Whitney U-test.

The results of the performance on the cognitive tests are provided in Table 2 . The scores of the groups were statistically similar. These findings suggest homogeneity of results across the three groups, that is, from a statistical standpoint, there were no notable differences in cognitive performance among the groups at the baseline assessment.

**Table 2:** Cognitive performance of the study sample

Variables	Group	Mean	±SD	Minimum	Median	Maximum	*p-value*
ACE-R	TG	89.97	±5.78	70.00	91.00	98.00	0.613ª
	ACG	90.14	±6.95	47.00	91.00	98.00	
	PCG	90.96	±4.80	73.00	92.00	100.00	
SKT	TG	3.45	±2.11	0.00	3.00	9.00	0.322ª
	ACG	3.49	±2.55	0.00	3.00	12.00	
	PCG	3.14	±2.42	0.00	2.00	13.00	
FAS: F	TG	14.46	±3.96	4.00	14.00	24.00	0.859ª
	ACG	14.59	±4.03	7.00	15.00	23.00	
	PCG	14.35	±3.64	7.00	14.00	26.00	
FAS: A	TG	13.17	±3.71	5.00	13.00	22.00	0.742ª
	ACG	13.57	±3.71	4.00	14.00	22.00	
	PCG	13.20	±4.17	4.00	13.00	24.00	
FAS: S	TG	13.65	±3.47	4.00	14.00	20.00	0.372ª
	ACG	12.97	±3.57	5.00	13.00	22.00	
	PCG	13.62	±4.08	4.00	14.00	26.00	
DSF	TG	7.97	±2.11	5.00	8.00	14.00	0.364ª
	ACG	8.54	±2.94	4.00	8.00	15.00	
	PCG	8.65	±2.69	4.00	8.00	14.00	
DSB	TG	5.54	±1.98	2.00	5.00	11.00	0.376ª
	ACG	5.35	±1.92	2.00	5.00	14.00	
	PCG	5.78	±1.97	2.00	6.00	11.00	
SSF	TG	7.65	±1.53	5.00	8.00	11.00	0.537ª
	ACG	7.24	±1.94	4.00	7.00	11.00	
	PCG	7.35	±1.84	3.00	8.00	12.00	
SSB	TG	6.26	±1.91	2.00	6.00	12.00	0.716ª
	ACG	6.44	±1.95	2.00	6.00	11.00	
	PCG	6.51	±2.05	2.00	6.00	11.00	
DSST	TG	59.15	±16.70	26.00	59.00	96.00	0.941ª
	ACG	58.35	±14.53	17.00	58.00	85.00	
	PCG	58.75	±14.93	8.00	59.00	93.00	
Letter number sequencing	TG	8.38	±2.07	4.00	8.00	14.00	0.786ª
	ACG	8.25	±2.44	3.00	8.00	13.00	
	PCG	8.52	±2.54	2.00	8.00	14.00	
TMT A: time	TG	45.18	±14.37	21.00	44.00	90.00	0.320ª
	ACG	49.53	±16.86	24.00	45.07	105.00	
	PCG	47.58	±17.70	24.00	43.06	118.00	
TMT A: errors	TG	0.26	±0.57	0.00	0.00	2.00	0.843ª
	ACG	0.25	±0.76	0.00	0.00	5.00	
	PCG	0.29	±0.70	0.00	0.00	3.00	
TMT B: time	TG	109.46	±66.10	51.00	100.00	471.00	0.137ª
	ACG	111.87	±44.21	47.00	107.00	221.00	
	PCG	102.23	±68.86	52.00	84.00	595.00	
TMT B: errors	TG	0.94	±1.31	0.00	0.00	6.00	0.928ª
	ACG	1.21	±1.73	0.00	0.00	7.00	
	PCG	1.03	±1.68	0.00	0.00	10.00	
Abacus calculation test (addition; time/number of right answers)	TG	50.42	±31.46	0.00	42.80	183.25	0.515ª
	ACG	51.23	±38.87	14.88	42.00	236.00	
	PCG	44.70	±24.08	10.17	39.17	139.60	

Abbreviations: ACE-R, Addenbrooke's Cognitive Examination-Revised; ACG, active control group; DSB, Digit Span Backward tests; DSF, Digit Span Forward tests; DSST, Digit Symbol Substitution Test; FAS, F-A-S Phonemic Verbal Fluency test; PCG, passive control group; SD, standard deviation; SKT, Syndrom Kurztest (Short Cognitive Test Performance); SSF, Spatial Span Forward test; SSB, Spatial Span Backward test; TG, training group; TMT A, Trail-Making Test A; TMT B, Trail-Making Test B.

Note: ^a^ *p* -value from the Kruskal-Wallis test.

No significant group differences were found regarding the psychosocial instruments, except for the GDS, in which the TG scored higher on depressive symptoms than the other groups ( Table 3 ).

**Table 3:** Sample characteristics for performance on psychosocial variables

Variables	Group	Mean	±SD	Minimum	Median	Maximum	*p-value*
CFI	TG	3.97	±2.68	0.00	3.50	11.00	0.114ª
	ACG	3.10	±2.09	0.00	2.50	8.00	
	PCG	3.11	±2.29	0.00	3.00	11.50	
DASS	TG	19.35	±13.44	0.00	16.00	62.00	0.069ª
	ACG	15.59	±11.19	0.00	14.00	52.00	
	PCG	14.10	±9.06	0.00	12.00	48.00	
DASS: stress	TG	11.29	±6.75	0.00	10.00	34.00	0.079ª
	ACG	9.49	±6.29	0.00	8.00	22.00	
	PCG	8.86	±5.86	0.00	8.00	32.00	
DASS: anxiety	TG	4.40	±5.04	0.00	4.00	26.00	0.108ª
	ACG	3.08	±4.25	0.00	2.00	26.00	
	PCG	2.71	±2.79	0.00	2.00	12.00	
DASS: depression	TG	3.66	±4.56	0.00	2.00	26.00	0.139ª
	ACG	3.02	±4.57	0.00	2.00	26.00	
	PCG	2.53	±3.69	0.00	2.00	18.00	
GDS	TG	2.88	±2.07	0.00	3.00	9.00	0.047ª
	ACG	2.40	±1.92	0.00	2.00	9.00	
	PCG	2.00	±1.55	0.00	2.00	6.00	
CASP-19	TG	42.85	±7.38	23.00	44.00	56.00	0.823ª
	ACG	43.56	±7.27	28.00	44.00	57.00	
	PCG	43.76	±7.08	27.00	43.00	57.00	
CASP-19: control	TG	9.55	±1.56	6.00	10.00	12.00	0.300ª
	ACG	9.81	±1.58	5.00	10.00	12.00	
	PCG	9.97	±1.46	5.00	10.00	12.00	
CASP-19: autonomy	TG	10.89	±2.02	7.00	11.00	14.00	0.469ª
	ACG	11.16	±2.17	5.00	11.00	15.00	
	PCG	11.29	±2.21	6.00	11.00	15.00	
CASP-19: pleasure	TG	12.49	±2.59	5.00	13.00	15.00	0.728ª
	ACG	12.46	±2.26	6.00	13.00	15.00	
	PCG	12.42	±2.08	8.00	12.00	15.00	
CASP-19: self-realization	TG	9.91	±2.82	3.00	10.00	15.00	0.936ª
	ACG	10.13	±2.89	5.00	10.00	15.00	
	PCG	10.08	±3.00	3.00	10.00	15.00	
MMROI (number of registers)	TG	29.26	±16.57	4.00	27.00	100.00	0.120ª
	ACG	24.60	±12.65	6.00	22.00	69.00	
	PCG	25.92	±14.50	3.00	24.00	94.00	

Abbreviations: ACG, active control group; CASP-19, Control, Autonomy, Self-realization and Pleasure scale; CFI, Cognitive Function Instrument; DASS, Depression, Anxiety and Stress Scale; GDS, Geriatric Depression Scale; MMROI, Minimum Map of Relationship of Older Individuals; PCG, passive control group; SD, standard deviation; TG, training group.

Note: ^a^ *p* -value from the Kruskal-Wallis test, followed by post hoc multiple comparisons when *p* < 0.05, in GDS (TG ≠ PCG).

## DISCUSSION

The objective of the present study was to describe the design of the Supera Cognitive Stimulation Study clinical trial and report the initial results collected at baseline. The results revealed that the different groups were similar in terms of sociodemographic, mood, cognitive, and functional variables. In randomized clinical trials, baseline evaluation is a key stage of the investigation. 4 The initial assessment is performed to identify whether there is any significant difference between the groups to be compared that may influence outcomes. The results of the present study suggest the randomization strategy adopted was effective.

The use of multicomponent strategies for cognitive stimulation in older adults has been the focus of previous studies using different intervention times, strategy types and follow-up intervals to measure maintenance of effects, as outlined in the summary by Brum and Yassuda. 37 Although their methodologies differed, these studies typically comprised only two groups: an experimental group and a control (generally passive) group. This highlights the need for studies in the field of cognitive stimulation involving older subjects that include the use of an active control group. This approach ensures a valid comparison between the effects of cognitive stimulation interventions and those of other activities or alternative interventions, helps control confounding variables which may influence study outcomes, improves the interpretation of results, and makes the study findings more generalizable for real-world situations. 4

Lira et al. 38 investigated the effects of a cognitive stimulation program on healthy older adults. The sample comprised 32 older individuals, stratified into an experimental group and a control group. The two groups were homogenous regarding age, gender, and level of schooling on the baseline tests, and no differences in performance were found. After 10 sessions (of 120 minutess each) of cognitive stimulation, the cognitive intervention group presented better performance in terms of language and cognitive functions, as well as fewer memory complaints compared with the control group.

Gómez-Soria et al. 4 examined the effect of cognitive stimulation on older individuals, focusing on general cognitive functioning and specific cognitive domains. The results have revealed significant improvements in general cognitive functioning, memory, orientation, praxis, and calculus. Moreover, evidence suggests that traditional or computer-based cognitive stimulation programs, with sessions of 45 minutes or longer, can be more effective for memory improvement memory. Reports suggest that participants aged up to 75 years benefit the most from cognitive stimulation in several aspects of cognition. These findings have important implications for the clinical practice, underscoring the importance of personalized/adapted cognitive stimulation for optimal improvements in cognitive function in older adults.

Employing a different approach, another study 5 explored the effects of cognitive stimulation on older individuals, focusing on psychosocial results, such as quality of life, activities of daily living, mood (depression and anxiety), self-esteem, and loneliness. The cognitive stimulation interventions presented a significant association with higher quality of life in healthy older participants. However, the results suggest that cognitive stimulation appeared to have no significant impact on the levels of anxiety and depression or activities of daily living. Nevertheless, the study 5 also addressed issues related to the duration and format of cognitive stimulation programs, such as the efficacy of cognitive stimulation used in different cultural contexts and in combination with pharmacological treatment.

Regarding the present study, the test results showed similar performance across all 3 groups of participants. Moreover, although a change in the level of depressive symptoms was evident, this was not accompanied by any significant group differences in terms of quality of life or cognitive functions. The limitations to the current study included the gender bias of the sample, which contained a greater proportion of female than male subjects (73.43% versus 26.57% respectively). This gender imbalance, together with the strict inclusion and exclusion criteria, may limit the generalization of the current results to future clinical trials involving a large sample of older adults. Another limitation was the use of self-reported exclusion criteria, which may have affected sample representativeness. Additionally, the Braztel-MMSE tool was used to determine the cut-off to exclude individuals with possible dementia. However, the sample had a level of schooling higher than that of the original validation sample, potentially including individuals with mild cognitive impairment whose symptoms may have been masked. This limitation should be considered when interpreting the results.

Therefore, the results obtained at study baseline serve as key indicators that should be considered in subsequent statistical analyses to guarantee the validity of the results in subsequent stages of the study. Moreover, it is important to note that the results on the different tests applied, such as the FAS and TMT, showed no statistical difference among the groups analyzed.

The designs of studies on neuroplasticity show this to be fertile ground for basic research, in that it yields vital information on human aging. In summary, this evidence shows that cognitive interventions to improve performance on mnemonic tasks are important for the functioning of older individuals, such as remembering to take medications, paying bills, and preparing balanced meals, thereby contributing to independence and reducing the risk of institutionalization and mortality.

Future studies should acknowledge the baseline assessment as a fundamental step in guaranteeing the validity of results. Lastly, similar studies should be conducted involving larger, more diverse samples and employing other measures, such as analysis of biological markers in clinical trials involving cognitive interventions.
